# Factors affecting pharmacology learning in integrated PBL in diverse medical students: a mixed methods study

**DOI:** 10.1186/s12909-024-05289-2

**Published:** 2024-03-21

**Authors:** S. A. Nicolaou, I. Televantou, A. Papageorgiou, A. P. Albert, A. W. Hitchings, P. McCrorie, Persoulla Nicolaou

**Affiliations:** 1 University of Nicosia Research Foundation, Nicosia, Cyprus; 2 Department of Life and Health Sciences, University of Nicosia, Nicosia, Cyprus; 3 Department of Educational Sciences, European University, Nicosia, Cyprus; 4 Department of Primary Care and Population Health, University of Nicosia Medical School, Nicosia, Cyprus; 5 Vascular Biology Section, Cardiovascular & Genetics Research Institute, St George’s, University of London, London, UK; 6 Institute of Medical, Biomedical and Allied Education, St George’s, University of London, London, UK; 7 St George’s University Hospitals NHS Foundation Trust, London, UK; 8 Department of Basic and Clinical Sciences, University of Nicosia Medical School, 2417 Nicosia, Cyprus

**Keywords:** Problem-based-learning, Basic science education, Personal characteristics/attitudes, Pharmacology, Prescribing, Mixed-methods

## Abstract

**Introduction:**

Problem-based learning (PBL) was introduced to address passive teaching limitations. However, it is not fully characterised as a teaching modality in pharmacology. The present study investigated the factors affecting pharmacology learning in an integrated PBL-based curriculum in diverse learners.

**Methods:**

Year 1 undergraduate medical students from two cohorts at St. George’s University of London and University of Nicosia, participated. Statistical analysis of pharmacology knowledge scores, at the beginning (pre-test) and end of the academic year (post-test), investigated readiness to benefit from PBL based on diverse student characteristics (educational background, age, gender, country of origin, ethnicity, native language, PBL experience). Focus groups/interviews and a survey investigated aspects of integrated PBL impacting learning in depth.

**Results:**

Pre- and post-test scores were positively correlated. Students with biomedical sciences degrees performed better at the pharmacology pre- and post-tests, while post-graduate degree holders performed better only at the pre-test. Effect size was of moderate magnitude. However, progress in learning (post-test performance after controlling for pre-test scores) was unaffected. Qualitative analysis revealed three major themes: 1) PBL as a learning environment; 2) PBL as a learning environment in pharmacology; and 3) PBL as a learning environment and confidence in prescribing. Under theme one, skill development, knowledge acquisition through collaboration and self-directed learning, group dynamics and preferred teaching methods were discussed. Under theme two, contextual learning, depth of knowledge and material correctness were raised. Under theme 3, students expressed variability in prescribing confidence. They perceived that learning could be improved by better integration, further references earlier on, more lectures and PBL facilitators with greater content expertise. The survey findings were consistent with those from focus groups/interviews.

**Conclusion:**

Pharmacology learning in a PBL-based curriculum is facilitated by constructive, collaborative and contextual learning. While baseline pharmacology knowledge may be advantageous, the other aforementioned characteristics studied may not affect readiness to benefit from PBL. However, further instructional scaffolding is needed, for example through further resources, lectures and self-assessment. The results from our study can inform evidence-based curriculum reform to support student learning further. Addressing learning needs could ultimately contribute to reducing medication errors through effective training of future prescribers.

**Supplementary Information:**

The online version contains supplementary material available at 10.1186/s12909-024-05289-2.

## Background

### Challenges in preparing medical students to be safe and competent prescribers

Medication errors are a worldwide public health burden [1, 2]. They compromise patient safety [3] and increase healthcare costs [4]. Strikingly, the majority of medication errors may be preventable [2, 5, 6]. The World Health Organization (WHO) highlights that lack of training in therapeutics and inadequate drug knowledge are contributing factors to medication errors [1]. Consistently, scholars have raised concerns about the inadequacy of medical education in preparing students for prescribing [7–12], with graduates showing a poor understanding of principles of drug action, adverse effects and drug-drug interactions [13]. Curriculum interventions that improve the learning of pharmacology in pre-clinical years may contribute to the development of prescribing skills in clinical years prior to graduation. The integrative nature of pharmacology [14–18] and the vast number of marketed drugs make teaching and learning pharmacology a challenge [11, 15, 19, 20]. Traditionally, pharmacology has been taught in isolation of other disciplines, using lecture-based learning (LBL). Educators have advocated the use of problem-based learning (PBL) to address the limitations of discipline-based learning and didactic teaching, which may promote a culture of passive learning [14, 15, 18, 19, 21–23]. Since its introduction in 1969 [23, 24], the use of PBL has expanded significantly around the globe [25, 26]. The pedagogical principles of contextual, collaborative and constructive learning, described below, provide a useful framework for exploring the beneficial effects of PBL.

### PBL educational framework and pedagogical principles

*Contextual learning.* PBL teaches traditionally-defined disciplines, such as anatomy, physiology and pharmacology, across organ systems (e.g. cardiovascular, respiratory etc.), where pertinent learning issues in each discipline are embedded within a healthcare problem. As such, the problem centres and integrates all learning around a professionally-relevant context to increase intrinsic motivation for learning [27]. The problem should be authentic, ill-defined and complex, with many unknowns and various possible solutions [27, 28]. Such problems can facilitate learners to compare and contrast tasks to enable them to deal with new problems in their future workplace [29]. *Collaborative learning.* Social theories of learning support the notion that social interaction promotes cognitive development [30]. In PBL, students work in small groups and the complex problem provides the starting point for high-order level collaborative interactions to enhance learning [31]. A PBL group represents a community of practice [32], whereby students have common learning goals and learn through participation and interaction. Group dynamics are complex and learning is affected by both cognitive and social aspects of group interactions [33]. Facilitators play an important role in enabling collaborative learning but should not act as traditional teachers. *Constructivism.* The principle of constructivism emphasizes the importance of learners as active seekers and co-creators of knowledge, based on learner experience [34, 35]. In this respect, PBL is consistent with constructivism since students are expected to activate their prior knowledge [36] and actively construct new knowledge through collaboration and self-directed learning [28]. Meaningful relationships between prior and acquired knowledge [37], elaboration and effective connections with the problem may have positive effects on long-term recall [38, 39]. Despite the wealth of information on PBL overall, it is not clear how students approach individual disciplines, including pharmacology, in integrated PBL. These pedagogical principles provided a useful lens for examining the effect of PBL-based learning in pharmacology in the present study.

### Effect of student diversity on learning in a PBL environment

Ideally, a pedagogical approach should cater to the needs of all learners regardless of their background. According to the aptitude-treatment interaction paradigm, students differ in their readiness to benefit from an educational methodology [40]. In the medical field, it is not currently clear which approach is best for whom, when and why [40], with only a few studies investigating the effects of student characteristics on learning in a PBL environment. For example, a meta-analysis reported that the effect of PBL on knowledge outcomes is affected by student expertise level [41]. There are no studies on specific disciplines, including pharmacology, addressing the preparedness of diverse learners to benefit from the PBL learning environment. In fact, studies investigating the effect of PBL have focused on overall knowledge or skills as outcomes and there is little information about how learners approach individual disciplines within an integrated PBL curriculum, such as pharmacology, and how their background may impact their learning. For example, students are expected to activate prior knowledge and build on it but no studies have investigated the impact of a student’s educational background on their learning. Even though PBL is based on adult learning principles, the effect of age has not been studied. Most recently, educational research has focused on the significance of gender and ethnicity as potential contributing factors to education disparities. For example, a recent study by Gaias et al, looked at educational intervention studies and showed that only 19% of empirical educational intervention studies and 6% of meta-analyses examined the potential effect of ethnicity [42]. The authors recommended that the design of educational studies also considers this aspect of diversity, especially for studies, which aim to improve student outcomes since they present an opportunity to reduce inequalities. The reasons behind the potential effect of ethnicity and gender are beyond the scope of this paper, however many articles are published in the literature, for example the aforementioned study by Gaias *et al* [42] and the more recent study by Gabriel [43]. Considering that many universities accept international students, it is important that educational studies also investigate the effect of students’ country of origin, for example due to different educational systems, and language on student learning for programmes delivered in a different language than their native one. Understanding the impact of student characteristics is important to allow educators to create an inclusive learning environment for diverse learners,

### Delivery of pharmacology in PBL curricula

Scholars have reported their personal or institutional experiences with the delivery of pharmacology in integrated PBL curricula [11, 14, 17, 19, 44–46]and in discipline-based PBL courses [21, 22, 47, 48], however there is paucity of data from well-designed research studies. In fact, empirical evidence of its effectiveness is sparse, since most studies are descriptive in nature. In the pre-clinical setting, the only study that investigated the efficacy of teaching pharmacology in an integrated, hybrid PBL curriculum was conducted in first- and second-year medical students in Indiana University [16]. It showed no significant difference in exam performance between students taught by PBL compared to LBL. However, PBL students had an increased positive experience of learning [16]. More recently, Brinkman et al, reported that integrated PBL in Years 2 and 3, alongside a course in Clinical Pharmacology and Therapeutics in Year 5, improved the prescribing competencies in final-year students, as compared to LBL [49]. However, these studies did not delve into how and why the PBL process and PBL-based curriculum impact learning. In fact, studies are inconclusive as to which components of the PBL process are most influential in student learning [50]. The present study aimed to shed light on how the PBL process impacts the learning of pharmacology in students of different backgrounds.

### Study objectives

The present study aimed to:

1. Investigate whether diverse student background characteristics (educational background and level of education, age, gender, country of origin, ethnicity, native language, admission test scores, experience with PBL) affect their readiness for pharmacology learning in an integrated PBL learning environment in first-year medical students. Pharmacology knowledge tests and quantitative methodology were used to address this aim.
2. Elucidate aspects of the integrated PBL-based medical curriculum, which impact student learning in pharmacology and confidence in prescribing. We used both qualitative methodology (focus groups/interviews) and a quantitative questionnaire that we have generated as part of this study to address this aim.

The findings of the present study can inform evidence-based curriculum changes to further address diverse learning needs. This could ultimately contribute to addressing the public health challenge of medication errors by improving training of future prescribers.

## Methods

### Participants and study design

**Fig. 1:**
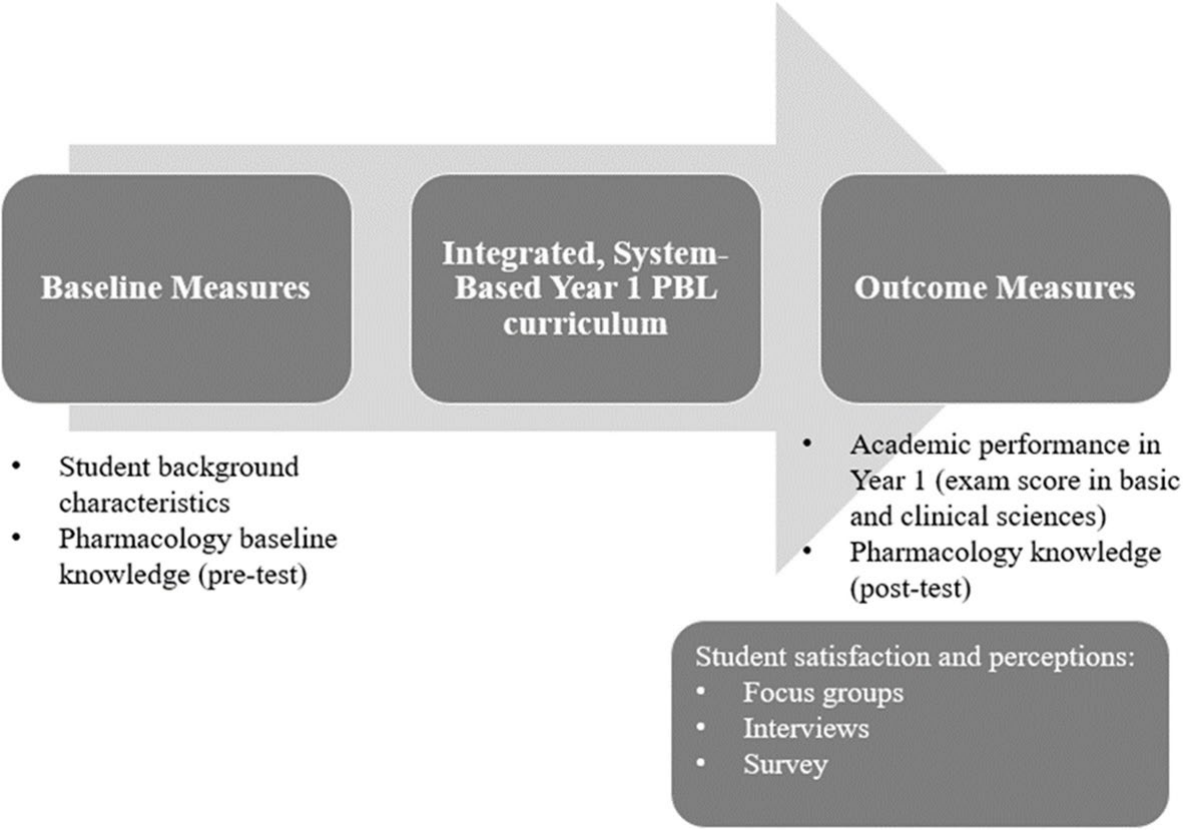
Study design. Baseline measures were recorded in the beginning of the year. Student characteristics included age, gender, country of origin, ethnicity, educational background (discipline and level of education), languages and admission test scores from standardized exams. Pharmacology baseline knowledge was assessed through a pre-test. Outcome measures recorded at the end of the academic year included performance in Year 1 examinations, in basic and clinical sciences, and pharmacology knowledge (post-test). Focus groups, interviews and a survey were used to obtain further insight into the student learning experience

The study design is shown schematically in Fig. 1. The study took place over two academic years, commencing in September 2019 and 2020. All Year 1 medical students of a four-year medical programme (MBBS), delivered concurrently at St. George’s University of London (SGUL) and University of Nicosia (UNIC), were invited to participate. The MBBS programme is a graduate-entry programme, which means that the minimum requirements for admission include an undergraduate degree. Therefore, all participants had completed, at minimum, a Bachelor’s degree. Completion of the MBBS programme leads to a primary medical qualification (undergraduate programme). Informed consent was sought from all participants. The inclusion of students from both institutions and two cohorts was important in increasing learner diversity. Students follow the same curriculum and are assessed using the same examinations in both institutions. The medical programme at UNIC has been quality-assured both by SGUL and the UK’s General Medical Council. The programme at UNIC is offered under a franchise agreement. The first two years of the MBBS course use an integrative, hybrid, system-based PBL, as its main didactic methodology. Year 1 starts with a Foundation module followed by six modules, which run sequentially, covering the following body systems/themes: 1) cardiovascular and respiratory systems; 2) gastrointestinal, renal and endocrine systems, 3) immune system, oncogenesis; 4) reproduction, growth and ageing, 5) skin and musculoskeletal systems and 6) nervous system. The learning week is centred around the PBL case, which brings together basic and clinical science learning relevant to the PBL case. Pharmacology is therefore delivered longitudinally, alongside other disciplines (e.g. anatomy, physiology), in all modules, with learning objectives incorporated, as they relate to the learning of the week. Learning in PBL is supported by a small number of lectures throughout the year. For example, in the asthma learning week in the cardiorespiratory module, students cover the following pharmacology learning objective: ‘Describe the mechanism of action, route of administration, place in therapy and major side effects of asthma treatments including short- and long-acting β_2_-agonists, corticosteroids, anti-muscarinic drugs, methylxanthine derivatives and leukotriene receptor antagonists’. Students cover the learning objective in the PBL sessions and learning is supported by a lecture on the management of acute and chronic asthma. The last two years are primarily spent in the clinical environment, with clinical teaching in all major specialties of medicine. Students gave their informed consent to participate in the study, and analysis was conducted using pseudonymized data.

### Participant characteristics

Participants were characterised at *baseline* with respect to their educational background (discipline and level of education attained), age, gender, country of origin, ethnicity, languages, experience with PBL and admission test scores from standardized exams (i.e. Medical College Admission Test (MCAT), delivered by the Association of American Medical Colleges, University Clinical Aptitude (UCAT), delivered by UK universities or Graduate Medical School Admissions Test (GAMSAT), developed by the Australian Council for Educational Research.

### Pharmacology knowledge

Pharmacology knowledge was assessed at *baseline (pre-test)* at the beginning of the year prior to the delivery of the curriculum and at the *end of the academic year (post-test),* i.e. after the curriculum was delivered, with a written paper comprising 50 single best answer (SBA) items [51], blueprinted against learning objectives in the Year 1 curriculum. SBAs are recognized to be more appropriate for the assessment of higher levels of knowledge, which are necessary for clinical practice [51]. As such, SBAs were constructed to assess application, rather than recall, of knowledge. Specifically, the questions were centred around a clinical scenario followed by a lead-in question and five alternate options, with one single best answer. For example, students were asked to choose the most appropriate drug for the patient in the scenario or choose the best description for a drug’s mechanism of action. Items were reviewed by pharmacologists, medical educators and a clinician to ensure clarity, factual correctness, appropriate level and conformance to SBA structure. Each SBA was worth one mark (test score range: 0–50). Reliability of the tests was estimated using the Kuder-Richardson 20 (KR_20_) coefficient.

The measures obtained at the *end of the academic year* included additionally:

- *Academic performance.* Academic performance at the end of Year 1 was defined as performance in the formal, integrated assessment of the MMBS programme in basic and clinical sciences, as assessed by two summative written exams consisting of 135 SBAs each. The exams assessed knowledge in anatomy, physiology, pharmacology, pathology, molecular biology and clinical sciences.
- *Student perceptions about aspects of the PBL process and curriculum that impacted their pharmacology learning* were assessed through qualitative methodology (*focus groups, individual interviews*) and quantitative methodolog*y* (completion of a *questionnaire* that was generated as part of this study).
  1. *Focus groups and interviews*. As a first step, a literature review was conducted to identify factors that may affect learning in a PBL environment overall and in pharmacology, specifically. Focus group and interview guides were subsequently constructed. Students that consented to participate in the study were invited to participate in the focus groups and interviews. Two moderators and a notetaker were present for each 60-min focus group. The 30-min individual interviews were conducted by one interviewer. All focus groups and interviews were audio- or video-recorded.
  2. *Questionnaire*. To ensure the quality of the questionnaire generated, the systematic process recommended by the Association for Medical Education in Europe (AMEE) was followed [52], including literature review, focus groups/interviews and identification of themes. 5 survey items were generated under each of the three identified themes, i.e. yielding a survey of 15-items. This length was chosen since it can yield reliable results, without compromising response rates [53]. In constructing the questionnaire both positively- and negatively-phrased questions were used to enable assessment of participants' attention, comprehension of the questionnaire and detect response bias or inconsistency. Negatively-phrased questions can additionally help balance out the overall tone of the questionnaire thus preventing response bias that may occur if all questions are positively phrased. Face validity was established by a panel of basic and clinical pharmacologists, medical educators and a clinician. It was finalized after being pilot tested on the target population in the mode of intended delivery.

### Statistical analysis

Data were processed and analyzed using SPSS Statistics. Missing data were treated using pairwise deletion. Statistical analysis included descriptive statistics, specifically mean score and frequency analyses, to determine the students’ background characteristics. To investigate the relationship between student background characteristics on *absolute performance in the pharmacology knowledge tests*, we used independent samples t-test or correlation analysis, depending on the measurement scale of the independent variable. Effect size was calculated for statistically significant findings, using Cohen’s d. In investigating the effect of student background characteristics on students’ *relative performance in the pharmacology knowledge tests*, we tested individual effects, using Mixed Analysis of Variance (ANOVA).

### Qualitative data analysis

Qualitative analysis followed the General Inductive Approach, as described more extensively previously [54]. Focus groups and individual student interviews were recorded, transcribed verbatim and analysed using thematic content analysis [55]. Two researchers analysed the data independently looking for codes, categories and themes. Any disagreements were discussed until agreement was reached.

## Results

### Participants

One hundred forty seven students consented to participate (academic year 2019–2020: *n* = 74, 48.7%; academic year 2020–2021: *n* = 73, 51.3%), drawn from a total cohort of 296 (participation rate 49.7%). The student background characteristics are shown in Table 1. Participation rates were similar in the two institutions (UNIC: *n* = 71; 48.3%; SGUL: *n* = 76; 51.7%). 77 (59.7%) of participants were female and 51 (39.5%) were male. The mean age of students was 25.67 ± 4.48 years old. In regards to ethnicity, the majority of students were white (58.9%). The remaining students (41.1%) came from a wide range of ethnicities, including African-American/Black, East Asian, Hispanic/Latino, Middle Eastern and Southeast Asian. As such, for further statistical analysis, and considering the sample size in the study, students were grouped together. The majority of students (*n* = 88; 70.4%) had an undergraduate degree in biomedical sciences (e.g. biology, biomedicine, health studies), while 29.6% (*n* = 37) had a degree in another discipline (e.g. psychology, international studies, chemistry, history). 65 students (49.6%) completed undergraduate (Bachelor’s) degrees, while 64 students (50.4%) additionally completed post-graduate degrees (a Master’s and/or a Doctorate); graduate degree areas of study were all in biomedical sciences. In regards to country of origin, the majority of students (43.4%) originated from Great Britain. Non-British students (56.6%) came from a range of different countries, including Israel, the United States, Australia, New Zealand, Canada, Lebanon, Germany, Brazil, France, Ireland, Egypt, Nigeria, Poland, Romania, Spain, and Venezuela. As such, for statistical analysis, and considering the sample size of the study, non-British students were grouped together. Furthermore, most students were native English speakers (76.7%). When asked about PBL experience in previous degrees, the majority of students (*n* = 121, 93.8%) reported no previous experience. The small number of students (*n* = 8; 6.2%) that had prior PBL experience only reported a PBL approach in a few courses/modules from their previous degree(s). Considering the large difference in the sample size of the two groups (i.e. students with or without PBL experience), PBL experience was excluded from further statistical analysis. For the focus groups and interviews, a heterogeneous group of 25 students participated. Their characteristics were similar to those of the sample participating in the quantitative study (Table 1).

**Table 1:** Student characteristics

	Quantitative Study	Focus Groups and Interviews
**Educational Institution (*n, %)***		
UNIC	71 (48.3%)	12 (48.0%)
SGUL	74 (51.7%)	12 (48.0%)
Missing	2 (1.4%)	1 (4.0%)
**Gender (*n, %*)**		
Male	51 (39.5%)	13 (56.5%)
Female	77 (59.7%)	10 (43.5%)
Missing	19 (12.9%)	2 (8.0%)
**Age (Mean, SD)**	25.67 (4.48)	25.57 (4.60)
Range	20–45	20–40
Missing (*n, %)*	22 (15.0%)	2 (8.0%)
**Ethnic Background (*n, %*)**		
White	76 (58.9%)	15 (60.0%)
Other	53 (41.1%)	8 (32.0%)
Missing	22 (15.0%)	2 (8.0%)
**Educational Background (*n, %*)**		
Biomedical Sciences	88 (70.4)	19 (76.0%)
Other than Biomedical Sciences	37 (29.6%)	3 (12.0%)
Missing (*n, %)*	22 (15.0%)	3 (12.0%)
**Highest Level of Education attained (*n, %*)**		
Bachelor’s	65 (49.6%)	10 (40.0%)
Master's or Doctorate Degree	64 (50.4%)	13(52.0%)
Missing (*n, %)*	18 (12.2%)	2 (8.0%)
**Country of Origin (*n, %*)**		
Great Britain	56 (43.4%)	9 (36.0%)
Other	73 (56.6%)	14 (56.0%)
Missing	18 (12.2%)	2 (8.0%)
**Native Language (*n, %*)**		
English	99 (76.7%)	18 (72.0%)
Other	30 (23.3%)	5 (20.0%)
Missing (*n, %)*	18 (12.2%)	2 (8.0%)
**PBL Experience (n, %)**		
No experience in previous degree(s)	121 (93.8%)	22 (88.0%)
All modules/courses in previous degree(s)	0	0
Most modules/courses in previous degrees	0	0
Some modules in previous degree(s)	0	0
A few modules/courses in previous degree(s)	8 (6.2%)	1 (4.0%)
Only one module/course in previous degree(s)	0	0
Missing	18 (12.2%)	2 (8.0%)

*Abbreviations*: *UNIC* University of Nicosia, *SGUL* St George’s University of London

Percentages represent proportions based on valid answers in each question. Students participating in focus groups and interviews (*n* = 25) were part of those that participated in the quantitative parts of the study (*n* = 147). Of the total 25 students participating in the qualitative data collection, eight (32%), six (24%) and eight (32%) participated in three different focus groups, while three participated in individual interviews

### Performance on pharmacology knowledge tests

Reliability of the pre- and post-tests were estimated by KR_20_ to be 0.62 and 0.65, respectively. The average pharmacology knowledge pre- and post-test scores were 18.08 ± 5.02 and 28.90 ± 5.39 (mean ± SD), respectively (test score range: 0–50). The majority of students that were in the lowest quartile in the pharmacology knowledge test at the beginning of the academic year (64.3%) remained in the lowest-achieving quartile at the end of the year, failing to reach the level of their peers with a higher pharmacology knowledge baseline score (Table 2). Only 7.1% of students in the lowest quartile in the pharmacology knowledge pre-test moved into the highest achieving quartile in the post-test. As expected, the mean scores in the pharmacology post-test increased progressively from the first to the fourth quartile (mean ± SD): 1st: 24.6 ± 5.0; 2nd: 27.5 ± 4.6; 3rd: 29.0 ± 4.9; 4th: 33.3 ± 3.7 (Table 2).

**Table 2:**
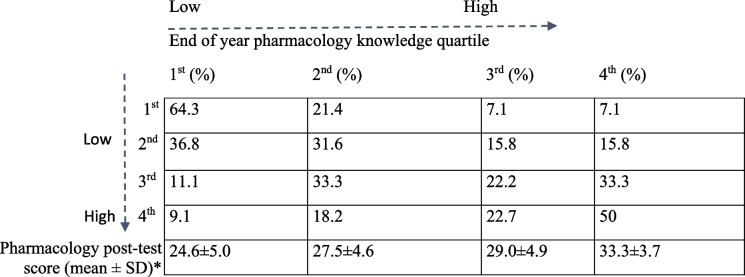
Student mobility between pharmacology knowledge levels during Year 1

Percentages refer to the proportions of students from each quartile in the pharmacology knowledge test at the beginning of the academic year that were in the corresponding quartile at the end of the academic year (*test score range: 0–50). Students starting off with lower pharmacological knowledge (i.e. in the 1st and 2nd quartiles) remained in the lowest quartiles, while students who had higher pharmacological knowledge continued to primarily stay in the highest performing quartiles, further supporting the significance of baseline knowledge. As expected, mean scores increased progressively in each of the quartiles (post-test mean scores shown in the last row)

### Effect of diverse student characteristics on pharmacology knowledge

A) Effect of student characteristics on absolute pharmacology knowledge test performance

**Fig. 2:**
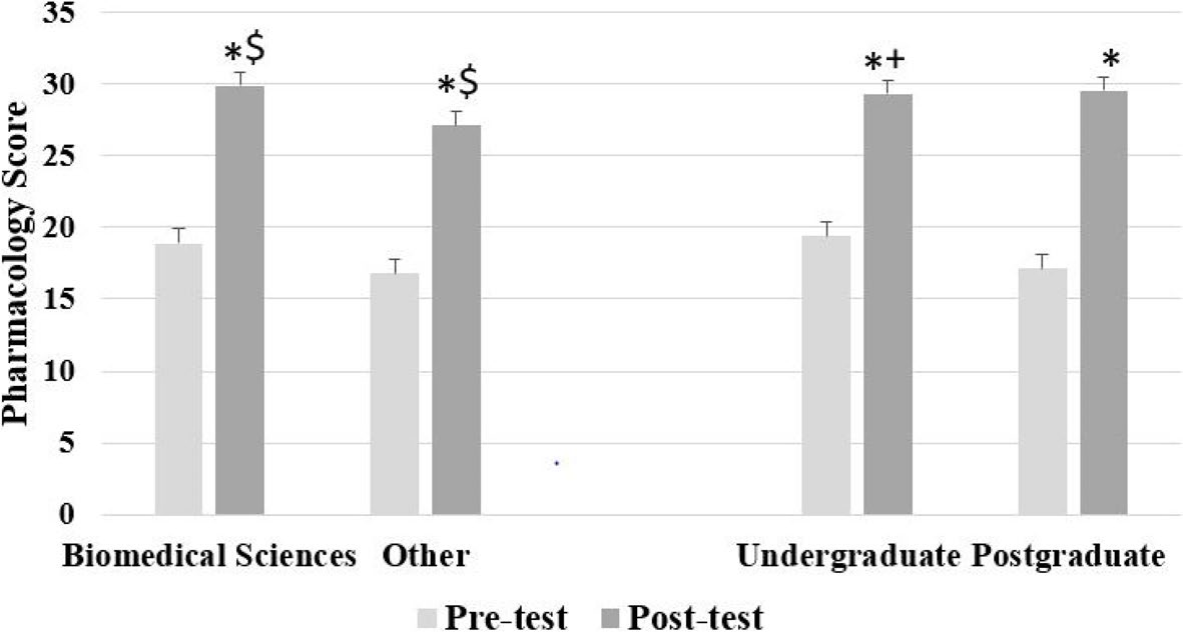
Achievement in pharmacology tests according to educational background. Students with a background in Biomedical Sciences performed significantly better both at the pre- and post-test (test range 0–50), as compared to those with no background in the Biomedical Sciences. Similarly, students with post-graduate degrees performed better at the pre-test but no difference was noted at the post-test, suggesting that all students benefited from the PBL curriculum independently of level of education. However, a background in Biomedical Sciences was advantageous in the learning of pharmacology at the end of the year. (*: *p* < 0.05: pre-test vs. post-test; $: *p* < 0.05: biomedical sciences vs. non-biomedical sciences in the same measurement; + : *p* < 0.05: undergraduate vs. post-graduate degree in the same measurement)

Pharmacology pre- and post-test scores were not related to educational institution, gender, ethnicity, country of origin, native language, age and admission test scores (Supplemental Material; Online Table 1). However, as shown in Fig. 2, students with a biomedical sciences background achieved higher scores in the pharmacology pre-test (mean ± SD, 18.85 ± 4.92) than their counterparts from different educational backgrounds (16.77 ± 4.46; *p* = 0.03, independent samples t-test). The difference was of medium magnitude, with Cohen’s d effect size being equal to 0.44. Similar results were seen in the pharmacology post-test (29.73 ± 5.14 *vs* 27.14 ± 5.50, respectively; *p* = 0.05). Similarly, Cohen’s d effect size revealed differences of medium magnitude (d = 0.50). Students with a post-graduate degree had an advantage in their pharmacology baseline knowledge (19.42 ± 4.25), as compared to students with no post-graduate degree (17.09 ± 5.68; *p* = 0.01) in the beginning of the academic year (Fig. 2). Cohen’s effect size was estimated to be d = 0.47, denoting a medium effect. However, there was no difference in performance at the pharmacology post-test score between students who had a post-graduate degree (29.33 ± 5.18) and those who did not (29.28 ± 5.59; *p* = 0.49). Furthermore, Cohen’s d was almost zero. Interaction graphs similarly showed that students with a biomedical sciences background continued to perform better at the end of the year (Online Fig. 1). However, the level of education attained was associated with progress in pharmacology, with students with an undergraduate degree making more progress than students with a graduate degree so that any differences in their pharmacology scores at baseline, were eliminated by the end of the year (Online Fig. 1).

### Performance in Year 1 and in the pharmacology knowledge tests

There was a significant positive correlation between academic performance in Year 1 and pharmacology knowledge, both at baseline (*r* = 0.46, *p* < 0.001) and at the end of the year (*r* = 0.40, *p* < 0.001). Performance in the pre-test was also positively correlated with performance in the pharmacology post-test (*r* = 0.52, *p* < 0.001). However, after controlling for the pharmacology pre-test score, academic performance in Year 1 explained no further the variability in students’ pharmacology post-test scores (R^2^ change = 0.06, *p* = 0.07).

B) Effect of student characteristics on relative pharmacology knowledge test performance

Pharmacology knowledge at baseline (pre-test score) explained 26.2% of the variability in the pharmacology post-test (F (1,53) = 18.84, *p* < 0.001, mixed ANOVA). Similar to our results for absolute performance above, relative performance in pharmacology was independent of educational institution (F (1,59) = 0.17, *p* = 0.68), gender ( F(1,56) = 0.55, *p* = 0.46), ethnicity (F(1,56) = 1.08, *p* = 0.30), country of origin (F(1,52) = 0.01, *p* = 0.92), native language (F(1,56) = 1.39, *p* = 0.24), age (F(1,54) = 0.31, *p* = 0.58) and admission test score (F(1,54) = 0.75, *p* = 0.39). Furthermore, an educational background in biomedical sciences (F (1,54) = 0.53, *p* = 0.47) and level of education attained (F (1,56) = 1.95, *p* = 0.17), did not have statistically significant relationships with relative performance.

### Focus groups and interviews

In 2019–20, one focus group was carried out at UNIC (*n* = 8) and two individual interviews with students at SGUL. In 2020–21, two focus groups took place one at each institution (*n* = 8, SGUL; *n* = 6, UNIC). The main themes, categories and codes that emerged from the qualitative data are summarized in Table 3. Those were: Theme 1—PBL as a learning environment, Theme 2—PBL as a learning environment in pharmacology, and Theme 3—PBL as a learning environment and confidence in prescribing. In Theme 1, students described the skills gained during PBL, the importance of learning with/from others and independently, perceptions about having tutors from varying backgrounds as well as the significance of group dynamics. Delving deeper into PBL and addressing pharmacology learning, in Theme 2, students highlighted the significance of the PBL case in providing a context for learning, facilitating the generation of pharmacology learning objectives and allowing consolidation of knowledge. Additionally, the implications for baseline pharmacology knowledge within the group were discussed. Finally, Theme 3 indicated that students in Year 1 have a low level of confidence in prescribing, particularly due to their limited exposure to drugs both in the PBL cases and in the clinical environment.

**Table 3:** Thematic content analysis from focus groups and interviews

Theme	Categories	Upper-level Codes with quotes
PBL as a learning environment	• Skills gained	• Critical thinking • Communication skills • Presentation ○ ‘*my ability to present now is far, far better’*
	• The PBL process	• Provides context (based-on real life) • Helps consolidate knowledge: ◦ ‘*I think PBL is useful for my learning, because it helps me understand better what I do and what I do not know…in the beginning of the week I may know nothing …I will go and build up on that during the week. … the end of the week I consolidate what I learned’* • Learning with/from others (teaching): ◦ ‘* when someone in the group explains something to you, this stays in your head, it does not go anywhere. … that is nice’* ◦ ‘*PBL is a critical component. I'm glad we have it’* • Self-Directed learning
	• The PBL tutor	• Tutor background ◦ ‘*…even with no science background may be really good facilitators’* ◦ *‘having a doctor as a first tutor was very helpful’* • Learning environment ◦ ‘*…the biggest role they play is to create that kind of environment where you feel comfortable asking questions and that its discussion is allowed’*
	• Group Dynamics	• Depth of knowledge pursued by group ◦ Other groups would focus on clinical aspect ◦ Group Dynamics: different experience depending on the background of the students. Students would be inclined to go into more depth on what they know ◦ Making sure that gaps are covered is group dependent • The functioning of the group ◦ Both are useful in learning and will be used later on as well. ‘Now we are learning and there's no right or wrong way of approaching it, as far as I'm aware, because we need both kinds of approaches’ ◦ Group Dynamics: the atmosphere/ chemistry of the group also matters. Will contribute more if you are more comfortable ◦ May engage in mature discussions ◦ May also pass on the wrong information if someone is assertive enough • Different groups ◦ *‘I have mostly positive experiences with different groupings’*
PBL as a learning environment in pharmacology	• The PBL case	• Drug learning objective generated • Linking the knowledge to the case is very helpful: ◦ *‘it is useful to go back to the case that we're actually looking at and we locate this is our patient.’*
	• Student Background	• Students with relevant background in pharmacology ◦ could be helpful in enriching learning or could hinder learning as the group relies on them
	• PBL tutor	• Tutor background ◦ *“expert content tutors and expert facilitators are the best for learning”*
	• Curriculum design	• Integration of PBL and lectures ◦ *‘we learn… from subjective reading, we get our resources, look at multiple sources and try to figure out what is right’* ◦ *‘Make pharmacology more coherent among the different PBL weeks. Pharmacology was a bit spread out,…harder to bring everything together’* • Level of knowledge ◦ *“Lecturers presume that we have this base level of knowledge”* ◦ *“Loading students with so much information in relation to drugs in Year 1 is overwhelming”* • Lack of prescribing • Spiral learning will help consolidate learning • Ways to improve the curriculum in pharmacology learning
	• The PBL process	• Learning from/with others ◦ *“great to go over things with people. It helps us find information on drugs.”* *“really good at putting things in perspective (drug and corresponding diagnosis).”*
PBL as a learning environment and confidence in prescribing	• Confidence in prescribing	• Lack of confidence • Increase confidence by graduation ◦ *‘ I do not think I would feel confident, because we only think of a medicine in certain settings… for right now, it sort of feels like difficult to understand how medicines can be used in different settings and for different reasons, other to the specific case scenario in the PBL’*
	• Increase confidence	• Incorporate the use of prescribing guidelines and more application of knowledge • Incorporate “an integrated pharmacology course” • More pharmacology lectures • Have quizzes at the end of each lecture to help students test and consolidate their learning (e.g. “Teaching is great but needs to be more interactive.”) • Review/practice sessions (e.g ◦ *“something to help determine the level of knowledge.”)* • Clinical placements • Mind maps • Application of knowledge ◦ *“Practice questions with explanations.”* • Better consolidation of knowledge (e.g ◦ *“initially provide a booklet for pharmacology to go back to’’* • Give drug options in PBL cases • Incorporate prescribing earlier and integrate prescribing in PBL (e.g. make report-back more of a discussion *‘so which drug would you use for this case, that kind of thing. So it's more of a problem-solving forcing you to like, apply your learning of that drug’* • Add prescribing workshops and reinforce application of knowledge (e.g. *…”to have patients with different conditions where you have to weigh up decisions like a really helpful way of applying, applying the knowledge.”*)

Students provided recommendations to improve the curriculum and support their pharmacology learning. They felt that pharmacology should follow a structure that *“…will make students feel safe in their knowledge and preparation for exams”*. To achieve that, they highlighted the need for better integration of PBL, lectures, clinical placements as well as additional resources earlier in the curriculum “*Integrate other resources in PBL e.g. Top 100 drugs – even though I understand that spiral learning is part of this.”* Lectures also featured prominently with students requesting a lecture at the end of the learning week. Finally, they asked for more guidance, to support their independent learning, on what is important to learn in pharmacology in Year 1 instead of having to guess *‘If we can have the level of knowledge that's expected of us at every stage you kind of know how deep you need to go’*.

Our qualitative results informed the generation of the questionnaire, which allowed us to corroborate our findings in more students, using quantitative methodology. The results are described below.

### Student questionnaire

As shown in Table 4, overall students agreed that PBL was beneficial for the development of independent learning skills (Question 2; mean ± SD, 3.80 ± 0.76; scale range 0–5 where 5 indicates full agreement) and communication and presentation skills (Question 3; 3.78 ± 0.90). There was also agreement that student diversity facilitated their learning (Question 5; 4.09 ± 0.71). However, students displayed neutral attitudes when rating their learning in PBL as compared to a lecture (Question 1; 3.47 ± 0.89). In regard to lectures in pharmacology, they agreed that they are the cornerstone of in-depth learning (Question 8 (reversely-coded); 2.21 ± 0.90) and they were neutral about lectures increasing their confidence in prescribing (Question 11; 3.44 ± 0.89). Students were not satisfied with the amount of pharmacology learning objectives (Question 9; 2.66 ± 0.79), the depth of pharmacology knowledge (Question 7 (reversely-coded); 2.57 ± 1.00) and its integration (Question 10 (reversely coded); 1.96 ± 0.66). They were neutral in regard to drug learning objectives enhancing their confidence in prescribing (Question 13; Mean = 3.07; SD = 0.85). However, students tended to agree that *‘Independent learning using evidence-based guidelines/sources increases [their] confidence in prescribing’* (Question 14; 3.57 ± 0.85). When it comes to tutors, students had neutral attitudes towards the relative importance of content expertise as compared to facilitation skills (Question 4 (reversely coded); 3.22 ± 1.10). However, there was agreement that *‘pharmacology learning is enhanced when there is a content expert in the room (tutor or fellow student)’* (Question 6; 3.92 ± 0.62). They were neutral in regard to the usefulness of clinical placements in facilitating competence in prescribing (Question 12 (reversely coded); 3.28 ± 0.73) but showed some agreement that upon graduation they will be competent prescribers (Question 15; 3.70 ± 0.73).

**Table 4:** Student questionnaire (*n* = 58)

	**Mean**	**SD**	Interpretation	
**PBL as a learning environment**				
1. I learn better in a PBL setting rather than in a lecture	3.47	0.89	Students displayed neutral attitudes when rating their learning in PBL as compared to a lecture	
2. PBL helps me develop my independent learning skills	3.80	0.76	Students agreed that PBL was beneficial for the development of independent learning skills	
3. PBL was not that helpful in developing my presentation and communication skills.^a^	3.78	0.90	Students agreed that PBL was beneficial for the development of and communication and presentation skills	
4. Regarding the PBL tutor, being a content expert is more important than being a good facilitator.^a^	3.22	1.10	Students had neutral attitudes towards the relative importance of content expertise as compared to facilitation skills	
5. Student diversity (different backgrounds and learning styles) facilitates my learning in the PBL environment	4.09	0.71	Students agreed that student diversity facilitated their learning	
**PBL as a learning environment in pharmacology**				
6. My pharmacology learning is enhanced when there is a content expert in the room (tutor or fellow student)	3.92	0.68	Students agreed that pharmacology learning is enhanced when there is a content expert in the room (tutor or fellow student)	
7. PBL-generated drug LOBs do not facilitate in-depth pharmacology learning.^a^	2.57	1.00	Students were not satisfied with the depth of pharmacology knowledge	
8. Pharmacology lectures are the cornerstone of learning pharmacology in depth.^a^	2.21	0.90	Students agreed that lectures in pharmacology are the cornerstone of in-depth learning	
9. I am satisfied with the amount of pharmacology LOBs generated in PBL cases	2.66	0.79	Students were not satisfied with the number pharmacology learning objectives in PBL	
10. I don’t feel that there is enough integration between the pharmacology taught in lectures and the pharmacology delivered in PBL.^a^	1.96	0.66	Students were not satisfied with the integration of pharmacology in PBL and lectures	
**PBL as a learning environment and confidence in prescribing**				
11. Pharmacology lectures increase my confidence in being able to prescribe	3.44	0.89	Students were neutral about lectures increasing their confidence in prescribing	
12. Clinical placements are not helpful in becoming a competent prescriber.^a^	3.28	0.73	Students were neutral in regard to the usefulness of clinical placements in facilitating competence in prescribing	
13. Drug LOBs generated in PBL enhance my confidence in becoming a competent prescriber	3.07	0.85	Students were neutral in regard to drug learning objectives enhancing their confidence in prescribing	
14. Independent learning using evidence-based guidelines / sources increases my confidence in prescribing	3.57	0.85	Students tended to agree that independent learning using evidence-based guidelines/sources increases [their] confidence in prescribing	
15. Upon completion of my MBBS course I believe I will be a competent prescriber	3.70	0.73	Students showed some agreement that upon graduation they will be competent prescribers	

Coding: (1 = Strongly Disagree, 2 = Disagree, 3 = Neither agree nor disagree, 4 = Agree, 5 = Strongly Agree)

^a^Reversely coded items (1 = Strongly agree, 2 = Agree, 3 = Neither agree nor disagree, 4 = Disagree, 5 = Strongly Disagree)

## Discussion

### Learning pharmacology in PBL

Scholars have reported some factors that may affect learning in pharmacology based on personal or institutional experiences with creating and delivering a horizontally- and vertically-integrated pharmacology curriculum in PBL medical programmes [14, 15, 17, 19, 45, 46]. However, there is a paucity of data from well-designed research studies investigating learners’ preparedness to benefit from PBL [40] and aspects of the PBL process affecting the learning experience [50], particularly from a student’s, rather than an instructor’s or medical educator’s, point-of-view. Our findings, from a heterogeneous student population, reveal the effect of educational background and provide insight into the factors that facilitate and hinder pharmacology learning. Specifically, our study highlights the significance of the PBL case in acting as a trigger for pharmacological concepts, the importance of student diversity and content expertise within the group, the implications of appropriate integration of pharmacology in the curriculum, including clinical placements, and the need for supporting student learning further through lectures, resources and self-assessment. Our findings can facilitate evidence-based curriculum reform and adaptations to PBL practice to support medical educators in preparing future prescribers.

### Implications for PBL theory and practice in diverse learners

#### Constructivism and prior knowledge

The students’ preparedness to benefit from PBL based on their background characteristics was determined by the pharmacology pre- and post-tests, which had acceptable reliability [56]. We found that students with higher baseline pharmacology knowledge in the beginning of Year 1 continued to outperform students with lower baseline knowledge at the end of the academic year. Our findings are consistent with the principles of constructivism and the prior knowledge activation theory, which postulates that prior knowledge is the foundation for new knowledge acquisition [39, 57]. Baseline knowledge may be particularly important for pharmacology considering its integrative nature, which is informed by many disciplines, for example biochemistry, physiology and pathophysiology [11, 17, 19].

In regards to educational background, we found that students with a biomedical sciences background or post-graduate degrees entered the medical programme with an advantage in their pharmacological knowledge. The effect size was moderate. Such degrees may in fact include teaching in pharmacology. Students with biomedical sciences backgrounds continued to outperform students with other degrees at the pharmacology post-test, albeit with a small difference. The size effect was however determined to be of medium magnitude. However, having a post-graduate degree did not affect pharmacology learning at the end of the year.

Progress in learning was not affected by any of the student characteristics investigated, including educational background and level of education attained. Even though two studies have investigated the effectiveness of a PBL medical curriculum in pharmacology learning [16, 49], to our knowledge, this is the first study to investigate the effect of baseline knowledge, educational background and other student characteristics on pharmacology learning in such a setting. However, studies conducted in non-PBL-based educational settings provide evidence for the significance of prior knowledge in pharmacology learning. For example, Hailikari and colleagues (2008) showed that performance of pharmacy students in the laboratory course of pharmaceutical chemistry was correlated with prior knowledge in four basic science courses that students previously took [58]. It is important that educators consider the needs of novice learners when designing and delivering the pharmacology curriculum, to create an inclusive learning environment that allows all students to benefit from the educational provision, independently of background.

### Collaborative learning

Consistent with social theories of learning [30], our qualitative data, combined with our results from the student questionnaire, suggest that collaboration and diversity in a group facilitated learning. The PBL collaborative process also allowed students to develop their communication and presentation skills. Group dynamics played a key role in the student’s learning experience, including the background knowledge of group members. As noted by students during the focus group discussions, they tended to focus more on areas they were more knowledgeable about. Furthermore, background knowledge also plays a role in group dynamics. During the focus group discussions, students suggested that a background in pharmacology might be helpful but they also stated that it could also be a hindrance as the group may rely too much on the one person with a pharmacology background to explore the pharmacology-related learning objectives.

### Contextual learning

According to the contextual learning principle, learning should preferably be centred around complex and authentic tasks in a professionally relevant context [28, 59], which can make the learning process more engaging. Our results support the usefulness of contextual learning. Students perceived the PBL case as providing a context, based on real life, which helped them put drugs in perspective, enabled the generation of drug learning objectives, and allowed consolidation of knowledge at the end of the learning week. Interestingly, a study by Pease and Kuhn suggested that, while social collaboration is important, the most effective component in the PBL process, is the problem itself [60]. Appropriate alignment of PBL cases in terms of pharmacology learning objectives with lectures and opportunities within the curriculum to apply and test pharmacology was important in the learning process.

### Confidence in prescribing and scaffolding in learning

Students perceived that PBL helped them develop independent learning skills and that using appropriate resources increased their confidence in prescribing. However, overall, students denoted lack of confidence in their prescribing skills, in agreement with a study in final year students, which showed moderate confidence in prescribing (2.9/5.0), as determined by the WHO 6-step self-rated confidence questionnaire in an integrated PBL curriculum [49]. It should be noted that students were neutral regarding the usefulness of clinical placements in facilitating competence in prescribing. This could reflect the extent of clinical placements in the pre-clinical curriculum, which is more limited, as compared to the clinical years, where training on clinical placements is the main curriculum delivery method. It is therefore important to further investigate confidence in prescribing, as well as competence in prescribing, and the usefulness of clinical placements as students progress in their medical studies since the study focused was focused on Year 1 students. At this early stage of their training, students called for further scaffolding to support their learning. In concordance, scholars have incorporated other mechanisms in hybrid delivery to support the delivery of pharmacology in PBL-based curricula, including lectures [14, 15], elective blocks [17, 19], assignment of resource persons for questions [17, 19], study guides [15], review sessions [15], essay and computerized modelling [14]. The need for scaffolding is widely acknowledged to diminish the level of complexity and promote transfer of knowledge to new problems [61, 62, 63]. This may be particularly important for novice learners, who lack a background in pharmacology, to support their self-directed learning in the early stages of learning [61, 64].

### Adaptations to PBL practice and evidence-based curriculum reform for pharmacology learning

The pedagogical principles, described above, provide a useful framework for considering adaptations to the teaching of pharmacology within integrated PBL medical curricula, based on the findings of the present study, to further support student learning in an inclusive learning environment. *Constructivism and prior knowledge.* The design of the pharmacology curriculum should consider the needs of students from different educational backgrounds. While understanding a student’s background knowledge is important overall, this is particularly important for pharmacology, which is a discipline that is informed by many other disciplines. An introductory module can be made available to support students with acquiring relevant prior knowledge that can serve as a foundation for learning pharmacology throughout the year so that they can further benefit from the PBL learning environment, independently of their background. A diagnostic pharmacology test may also be helpful to allow students to assess their own level of knowledge and identify areas to focus their learning during the first year of their studies. *Collaborative learning.* Our data suggest that this important aspect of learning in a PBL environment should be maintained while ensuring that the groups are diverse in nature with a mix of different student backgrounds. For the learning of pharmacology, it is useful to have a content expert in the group, this can either be the tutor or a student with a relevant background. *Contextual learning.* The PBL curriculum should carefully and systematically integrate pharmacology learning, wherever relevant. PBL cases should be compiled by multidisciplinary teams, which also include basic/clinical pharmacologists. This will ensure that the PBL case and the curriculum will draw out relevant pharmacological concepts in appropriate depth. Student learning can be further contextualized by building on the learning in the clinical environment through short clinical placements in Year 1. Learning in pharmacology should be explicit, and not implied, both in the PBL curriculum and when setting expectations for student learning in the clinical environment. *Scaffolding in learning.* Additional resources, in the form of reading material or study guides, should be provided to students to support them in focusing and prioritizing their learning throughout the year. Lectures may also be added in curricula, possibly, after self-directed learning, to reinforce the main learning points in hybrid PBL programmes. Formative weekly quizzes can be made available to allow students to monitor their learning, receive feedback and seek support, as needed, from the responsible academics. The quizzes or other assignments should allow students to apply their knowledge to clinically-relevant settings, that can increase their confidence in prescribing.

### Limitations

While all Year 1 students from two different cohorts were invited to participate, the response rate was moderate, with about half of the students participating. The number of participants may present limitations for statistical analysis, however statistically significant effects were noted with our study sample. In regards to the effect of language, proficiency was not examined, which could have provided further insight into the potential effect of language. Furthermore, due to the Covid-19 pandemic, part of the curriculum, particularly in 2019–2020, was delivered utilizing online methodology, including online PBL tutorials. The content of the curriculum and process for PBL were overall unchanged and the results from both cohorts (i.e. in 2019–2020 and 2020–2021) were consistent, suggesting that the mode of delivery did not impact the results. Our study presented findings from one PBL curriculum. However, the curriculum was delivered concurrently at two different sites in SGUL and UNIC, with similar results. Additionally, our findings are consistent with those from other medical schools delivering hybrid PBL curricula, based primarily on descriptive studies, supporting the generalizability of our results [11, 14, 15, 17, 19, 29–31].

## Conclusions

Our results highlight the significance of the PBL case, the importance of appropriate integration of pharmacology in the curriculum, the role of content experts within the PBL group and the benefits of learning with/from others. Our findings suggest that pharmacology learning in a PBL-based curriculum is facilitated by constructive, collaborative and contextual learning. Still, there is a need for further instructional scaffolding in an integrated PBL setting, for example through lectures, resources and quizzes. This may be particularly important for students who lack a background in pharmacology. Further longitudinal studies can shed further light on the effect of PBL-based learning in diverse learners on the development of prescribing skills. Addressing student learning needs early in their studies through evidence-based curriculum interventions could ultimately contribute to reducing medication errors through effective training.

### Supplementary Information

**Supplementary Material 1.**

**Supplementary Material 2.**

## Data Availability

The datasets used and analysed during the current study are available from the corresponding author on reasonable request.

## Abbreviations

PBL: Problem-based learning
SGUL: St George’s University of London
UNIC: University of Nicosia
WHO: World Health Organization
LBL: Lecture-based learning
MBBS: Bachelor of Medicine and Bachelor of Surgery
MCAT: Medical College Admission Test
UCAT: University Clinical Aptitude
GAMSAT: Graduate Medical School Admissions Test
SBA: Single best answer
KR_20_: Kuder-Richardson 20
AMEE: Association for Medical Education in Europe
ANOVA: Mixed Analysis of Variance
