# Supplementary material for: Factors affecting pharmacology learning in integrated PBL in diverse medical students: a mixed methods study
Source: BMC Med Educ. 2024 Mar 21;24:324. doi: 10.1186/s12909-024-05289-2 (PMC10958917; doi:10.1186/s12909-024-05289-2)
Supplement: Supplementary file 1 — Supplementary Material 1. [file 12909_2024_5289_MOESM1_ESM.docx]

**ONLINE TABLE 1.** Impact of student background characteristics on students’ absolute pharmacology performance at pre-and post-test

|  | **Pharmacology Knowledge Pre-Test** | | | |  | **Pharmacology Knowledge Post-Test** | | | |
| --- | --- | --- | --- | --- | --- | --- | --- | --- | --- |
|  | **Mean (SD)** | **T** | **Df** | **P** |  | **Mean (SD)** | **T** | **df** | **p** |
| Educational Institution  UNIC  SGUL | 17.26 (4.71)  18.75 (5.24) | -1.57 | 108 | 0.12 |  | 28.00 (5.40)  29.64 (5.59) | -1.21 | 116 | 0.11 |
| Gender  Male  Female | 18.19 (4.33)  18.47 (5.61) | 0.28 | 96 | 0.78 |  | 29.54 (5.93)  29.15 (4.91) | -0.29 | 63 | 0.78 |
| Ethnic Background  White  Other | 18.61 (4.82)  17.95 (5.44) | -0.63 | 96 | 0.53 |  | 29.47 (5.59)  29.10 (5.00) | -0.28 | 63 | 0.78 |
| Educational Background  Biomed  Other | 18.85 (4.92)  16.77 (4.46) | -1.97 | 93 | **0.03^1^** |  | 29.73 (5.14)  27.14 (5.50) | -1.64 | 61 | **0.05^1^** |
| Level of Education  Bachelor’s  Master's or Doctorate | 17.09 (5.68)  19.42 (4.25) | -2.32 | 96 | **0.01^1^** |  | 29.28 (5.59)  29.33 (5.18) | -0.3 | 63 | 0.49^1^ |
| Country of Origin  Great Britain  Other | 19.24 (5.31)  17.70 (4.82) | -1.50 | 96 | 0.14 |  | 29.27 (5.60)  29.34 (5.05) | .05 | 63 | 0.96 |
| Native Language  English  Other | 18.28 (5.28)  18.54 (4.41) | -0.22 | 96 | 0.83 |  | 28.83 (5.25)  31.23 (5.23) | -1.48 | 63 | 0.15 |
| Continuous variables | **r** | **p** |  |  |  | **r** | **p** |  |  |
| Age | 0.07 | 0.50 |  |  |  | 0.15 | 0.25 |  |  |
| Admission test scores | 0.20 | 0.06 |  |  |  | 0.14 | 0.29 |  |  |

*Note. ^1^* The p-value reported is based on a one-tailed t-test.
