# Supplementary material for: Factors affecting pharmacology learning in integrated PBL in diverse medical students: a mixed methods study
Source: BMC Med Educ. 2024 Mar 21;24:324. doi: 10.1186/s12909-024-05289-2 (PMC10958917; doi:10.1186/s12909-024-05289-2)
Supplement: Supplementary file 2 — Supplementary Material 2. [file 12909_2024_5289_MOESM2_ESM.docx]

**
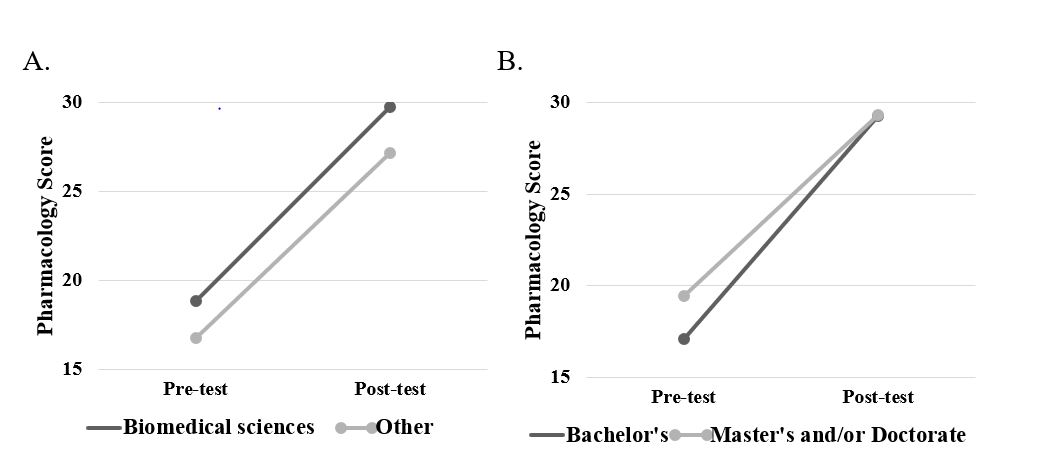
**

**Online Figure 1:** Interaction graphs for achievement in pharmacology tests, according to educational background. A) Students with a background in biomedical sciences outperformed students from other educational backgrounds at the beginning of the year and continued to perform better at the end of the year (test score range 0-50). B). However, students with no post-graduate degree reached the level of students with post-graduate degrees, that outperformed them in the beginning of the year.
